# Detection of superficial and buried optic disc drusen with swept-source optical coherence tomography

**DOI:** 10.1186/s12886-022-02447-2

**Published:** 2022-05-13

**Authors:** Xiaohong Guo, Yingjie Wu, Yuhong Wu, Hui Liu, Shuai Ming, Hongpei Cui, Ke Fan, Shuyin Li, Bo Lei

**Affiliations:** Henan Eye Institute, Henan Eye Hospital, Henan Branch of National Clinical Research Center for Ocular Diseases, People’s Hospital of Zhengzhou University, Henan Provincial People’s Hospital, Zhengzhou, 450003 Henan China

**Keywords:** Optic disc drusen, Swept-source optical coherence tomography, Fundus autofluorescence, Lamina cribrosa

## Abstract

**Background:**

To detect the superficial and buried optic disc drusen (ODD) with swept-source optical coherence tomography (SS-OCT).

**Methods:**

Retrospective cross-sectional study. Twenty patients (age 18–74 years) diagnosed with ODD via B-scan ultrasonography were analysed. All patients underwent color fundus photography (CFP), B-scan ultrasonography, fundus autofluorescence (FAF), and SS-OCT. We defined each hyporeflective signal mass of SS-OCT as an ODD, recorded its location and relationship with Bruch’s membrane opening (BMO), and other ophthalmic imaging characteristics.

**Results:**

Twenty (33 eyes) patients had 54 ODDs in all, except one eye did not show abnormal optic disc findings on SS-OCT. We classified ODD into three categories: ODD above BMO, ODD across BMO, and ODD below BMO. The ODDs across BMO were the largest, followed by ODDs below BMO, and those above BMO. The location of the ODDs: One (1.9%) was in the border tissue of Elschnig, 6 (11.1%) might span across the lamina cribrosa, 16 (29.6%) were above BMO located in the neuroepithelial layer, 9 (16.7%) spanned across BMO located near the center of the optic disc, 18 (33.3%) were below BMO located near the center of the optic disc, 4 (7.4%) were below BMO located within the optic disc rim. When the anterior margin was ≥ 100 μm from the BMO, clear autofluorescence could be seen.

**Conclusion:**

Multimodal imaging provided a deeper understanding of ODD. SS-OCT illustrated more details about the relationship between the posterior surface of ODD, BMO and the lamina cribrosa.

## Background

Optic disc drusen (ODD) are acellular deposits composed of calcium, amino acids, nucleic acids, and mucopolysaccharides. ODD are usually asymptomatic but over time can cause visual field defects [1, 2]. In addition, ODD may also be a high-risk factor for diseases such as anterior ischemic optic neuropathy (AION), retinal artery occlusion, or retinal vein occlusion. The incidence of ODD in the population is around 0.3%, with no significant difference between males and females. However, autopsy studies have found that the prevalence of ODD, which is about 2.4%, is much higher. The difference of the detected ODD percentage between clinical examination and autopsy inspection indicates that most ODD are not found in patients and their incidence may be overlooked significantly [1, 3].

The reason that a much less ODD patients were clinically diagnosed may attribute to the position of the ODD and the imaging techniques that applied. First, ODD are either superficial or buried. When located on the surface of the optic disc, they can be directly observed via ophthalmoscopy or fundus photography, appearing as an irregular mass in pale yellow color. However, when buried in the optic disc, they can be difficult to detect. Secondly, patients suspected having ODD are usually examined by B-scan ultrasonography or fundus autofluorescence (FAF). Nevertheless, these techniques exhibit low image resolution and provide limited information concerning ODD’s depth or internal characteristics.

In recent years, the emergence of optical coherence tomography (OCT) enables us to obtain the cross-sectional information of the retina which was not possible before. Especially, with a wavelength of 1050 nm, swept-source OCT (SS-OCT) presents high penetration and expands detection depth up to 6 mm. By combining SS-OCT and other imaging techniques, we explored the full spectrum of the morphological characteristics of ODD. We showed that multimodal image approach exhibited the imaging characteristics of the ODD not only for those located in the surface, but also for those buried deeply in the optic nerve head. Understanding the comprehensive profile of ODD would improve the clinical detection rate and pave the way for further understanding the mechanisms of the lesion.

## Patients and Methods

We performed a retrospective cross-sectional study about the multimodal imaging findings of ODD. We analyzed the patients diagnosed with ODD via B-scan ultrasonography at Henan Eye Hospital, Henan Provincial People’s Hospital (Zhengzhou, China) from October 2019 to December 2020. This study was performed in.

accordance with the tenets of the Declaration of Helsinki and approved by the Henan Eye Hospital (approval number HNEECKY-2021(04)). Also, we got the informed consent from all the participants.

There were 20 patients including 9 (45%) male and 11 (55%) female; 13 (65%) cases were bilateral and 7 (35%) unilateral, with a total of 33 eyes. Patient age range was 18 ~ 74 years (average, 39.2 years). Best corrected visual acuity (BCVA) was 0.2 ~ 1.0 (average, 0.65). Inclusion criteria were ODD patients diagnosed after two experienced neuro-ophthalmologists had comprehensively evaluated their medical history and examination results. Their B-scan ultrasonography results showed hyperechoic spots on the optic disc with sharp edges and acoustic shadowing. Exclusion criteria were systemic diseases that may affect optic nerve function, ocular trauma, or optic nerve diseases, including optic neuritis, glaucoma, AION, central retinal artery occlusion, central retinal vein occlusion, optic disc tumor and so on.

All patients underwent color fundus photography (CFP), B-scan ultrasonography, FAF, and SS-OCT. We performed CFP using a Canon fundus camera (CR-2 AF; Canon Medical Systems Corp., Tokyo, Japan). B-scan ultrasonography was conducted using a Meda scanner (MD-2400S; MEDA Co., Tianjin, China) with a 10-Hz probe and a gain of 50 dB. We obtained SS-OCT images using a SS-OCT (VG200D; SVision Imaging, Luoyang, Henan, China) with a laser wavelength of 1050 nm and an acquisition speed of 200,000 × per second. With the optic disc at the center, we obtained a horizontal and vertical volume scan of 6 × 6 mm^2^ (768 × 768).

**Fig. 1:**
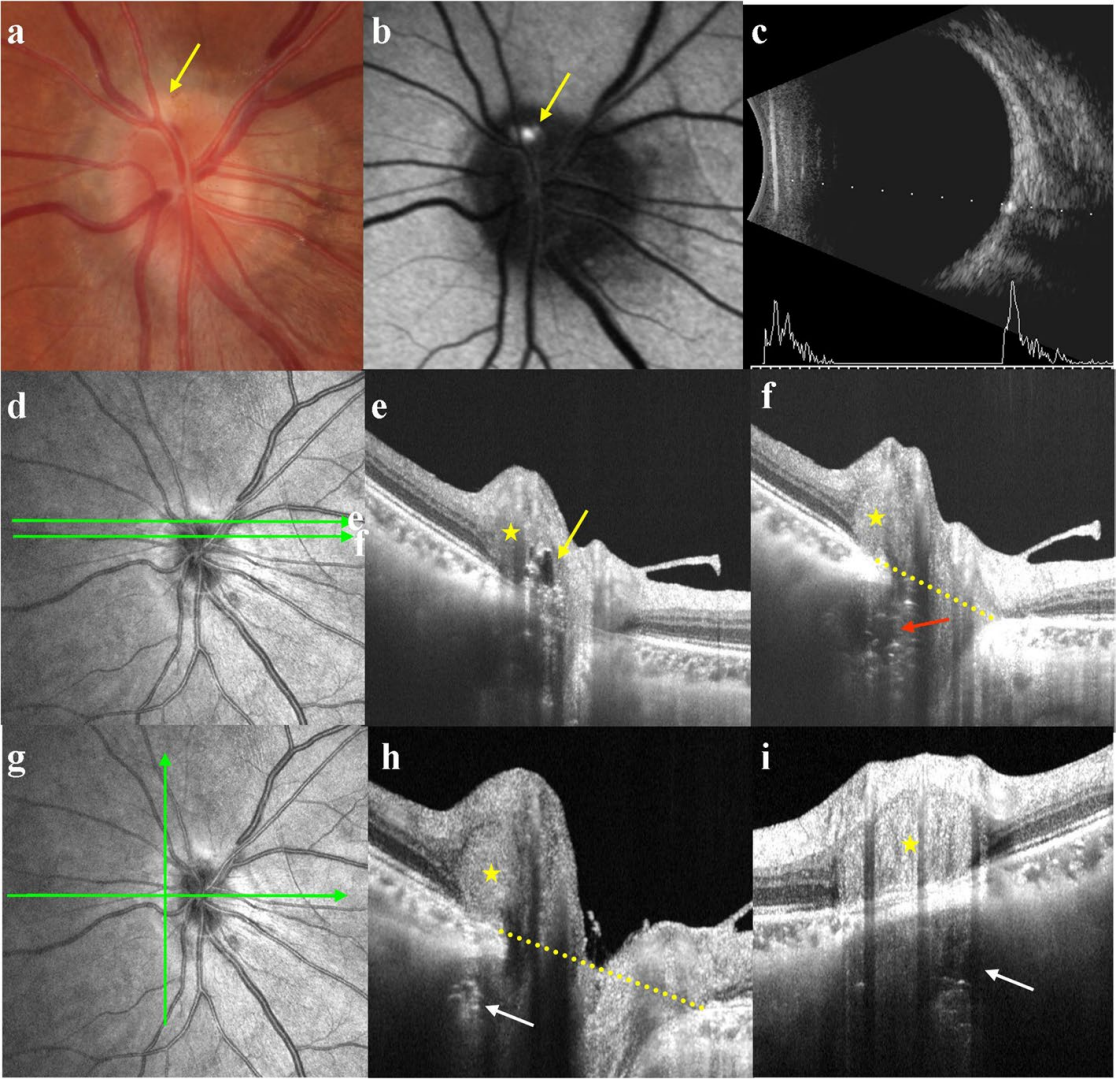
Multimodal imaging of a typical superficial ODD **a** CFP showed pseudopapilledema and a punctate pale-yellow deposits (yellow arrow). **b** FAF clearly showed autofluorescence. **c**: B-scan ultrasonography displayed strong echo spots with acoustic shadowing. **d** The upper scan line corresponded to **e** and the lower scan line corresponded to **f**. **e** The ODD was in the neuroepithelial layer of optic disc nasal superior (yellow arrow corresponding to yellow arrow in **a** and **b**). **f** The ODD was near the center of the optic disc (red arrow). **g** Horizontal scan line corresponded to **h**, vertical scan line corresponded to **i**. **h** and **i** ODD was in the border of Elschnig. The yellow stars in **e**, **f**, **h** and **i** indicated PHOMS, corresponding to pseudopapilledema. The yellow dotted line in **f**, **h** indicated the BMO. CFP = color fundus photography; FAF = fundus autofluorescence; PHOMS = peripapillary hyperreflective ovoid mass-like structure; BMO = Bruch’s membrane opening

Based on enhanced depth imaging OCT (EDI-OCT) or SS-OCT, ODD were defined as hyporeflective signal masses surrounded by hyperreflective margins in previous study [1, 4–6]. Since it is possible that not all patients have hyperreflective horizontal lines, we defined each hyporeflective signal mass as an ODD in this study. The number of ODD in each eye was recorded. ODD were scanned on the optic disc horizontally and vertically (Figs. 1d, g and 3d). Bruch’s membrane opening (BMO) was used as a reference plane (Fig. 1h, h, yellow dotted line). The vertical size was the distance between the anterior and posterior margins of the ODD and its relative position with BMO was measured. A margin laid above the BMO was positive and below the BMO was negative. The largest transverse diameter of the ODD in the horizontal direction was also measured.

FAF were captured using a Heidelberg retinal angiography (Spectralis HRA + OCT, Heidelberg Engineering, Heidelberg, Germany). FAF scanning was performed with a 488-nm laser (Blue-light FAF), using 30º × 30° field of view, optical resolution of 1,536 × 1,536 pixels. Three to five FAF images were collected by adjusting the sensitivity.

## Results

**Fig. 2:**
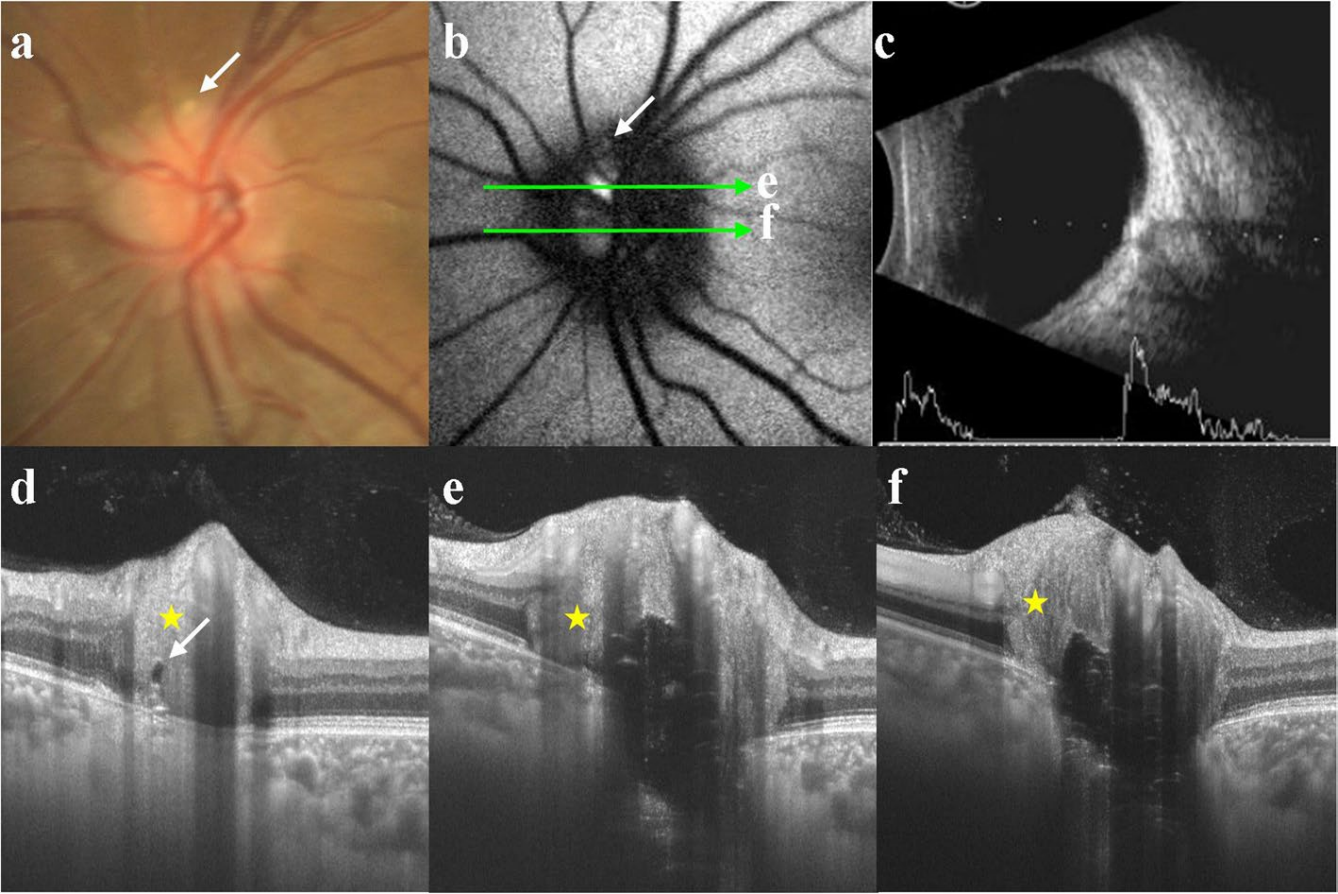
The relationship between ODD and AF with different depths **a** CFP shows pseudopapilledema and a punctate pale-yellow deposits (white arrow). **b** The weak autofluorescence (white arrow) corresponded to ODD in **a** and **d** (white arrows). Patchy autofluorescence was seen on the nasal side of the optic disc and the scan lines **e** and **f** corresponded to figures **e** and **f**, respectively. ODD in **e** was closer to the surface of optic disc than the ODD in **f**. Autofluorescence was brighter in **e** but weaker in **f**. **c** B-scan ultrasonography showed strong echo spot with acoustic shadowing. The yellow stars in **d**, **e** and** f** were PHOMS, corresponding to pseudopapilledema. CFP = color fundus photography; FAF = fundus autofluorescence; PHOMS = peripapillary hyperreflective ovoid mass-like structure

**Fig. 3:**
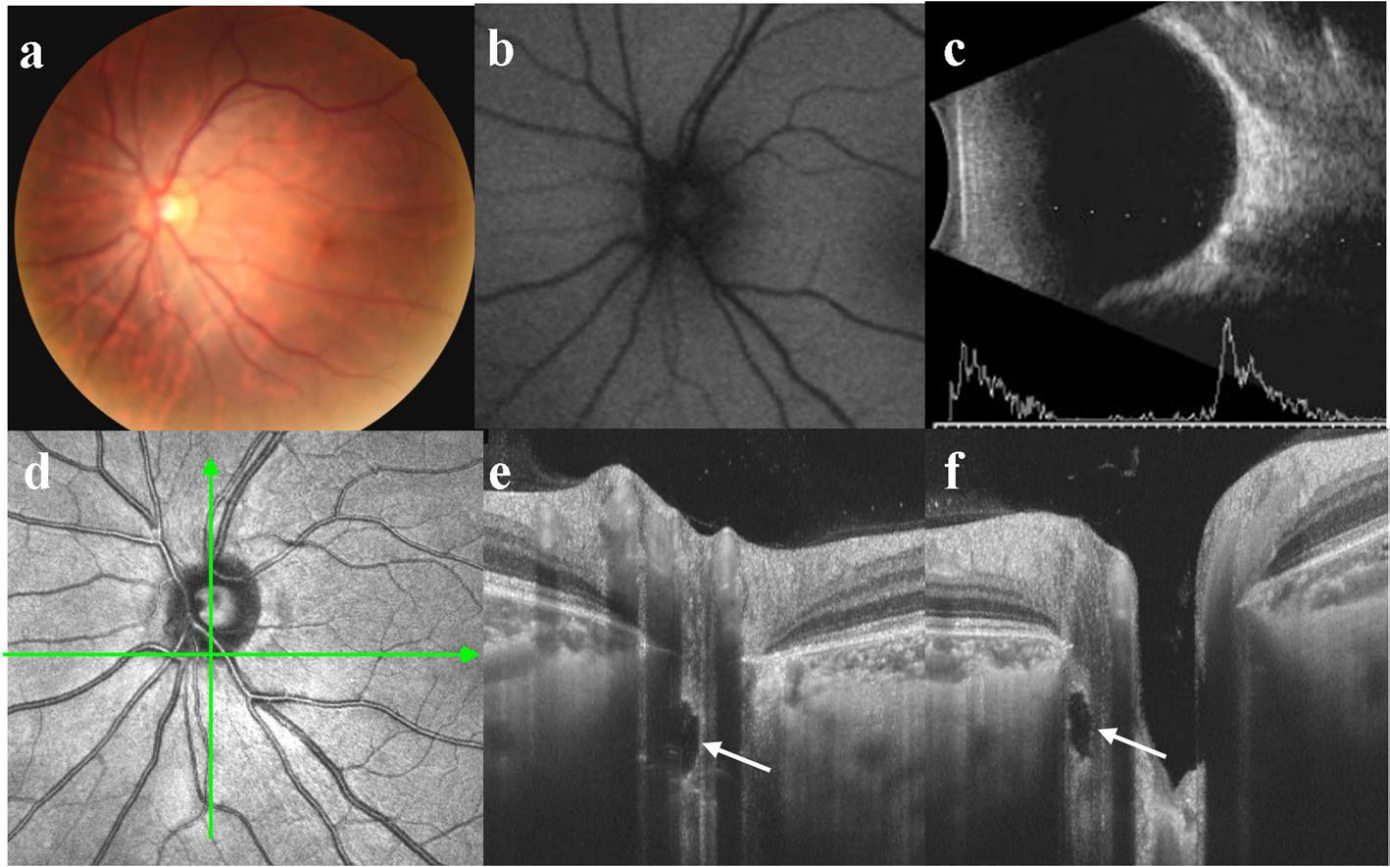
Multimodal imaging of a typical buried ODD **a** There was no noticeable pseudoedema in the optic disc. **b** No autofluorescence was detected. **c** B-scan ultrasonography showed strong echo spots with acoustic shadowing. **d** Horizontal scan line corresponded to **e**, vertical scan line corresponded to **f**. **e/f** An ODD was located near the rim of the optic disc (white arrow), and there was no highly reflective mass around the optic papilla

**Fig. 4:**
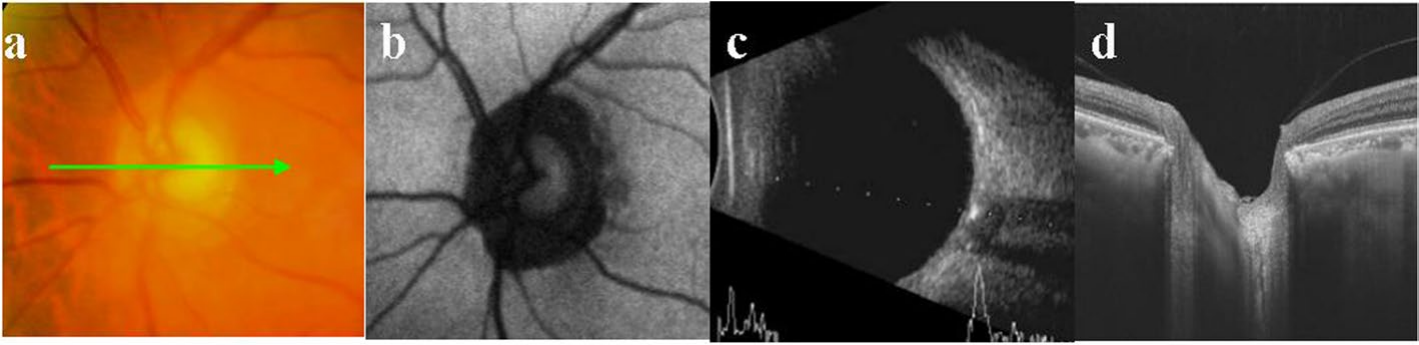
Multimodal imaging of a special buried ODD ODD was confirmed by B-ultrasound, however, no special finding was noticed in the fundus photography, no noticeable autofluorescence and abnormality was seen in SS-OCT

Of the 33 eyes, 9 (27.3%) had superficial ODD, which appeared as punctate pale-yellow deposits or mass (Figs. 1a and 2a), while 24 (72.7%) had buried ODD (Figs. 3a and 4a). 21 (63.6%) had pseudopapilledema (Figs. 1a and 2a), while 12 (36.4%) had no pseudopapilledema (Figs. 3a and 4a). All patients had strong echo spots with acoustic shadowing on the optic disc after decreasing gain (Figs. 1c, 2c, 3c and 4c).

### Imaging characteristics of ODD on SS-OCT

Morphology: Of the 33 eyes, 32 (97%) showed a typical hyporeflective signal mass surrounded by a hyperreflective margin with enhanced hyperreflective horizontal lines. In the remaining eye (3%), no abnormality was found in the optic disc (Fig. 4d). Of all 33 eyes, 21 (63.6%, including 9 with superficial and 12 with buried ODD) showed pseudopapilledema on CFP, and the corresponding SS-OCT showed a peripapillary hyperreflective ovoid mass-like structure (PHOMS; Figs. 1e, f, h, i and 2d, e, f, respectively). Pseudopapilledema and PHOMS were not seen in 12 eyes (36.4%, all were buried ODD, Fig. 3e, f).

Number: ODD existed alone or in clusters. Of all 33 eyes, 20 (60.6%) presented one ODD, 6 (18.2%) had two ODDs, 3 (9.1%) had three ODDs, 2 (6.1%) had four ODDs, 1 (3.0%) had five ODDs, and 1 (3.0%) had zero. Each eye had on average 1.7 ODD and the median was 1 per eye.

**Fig. 5:**
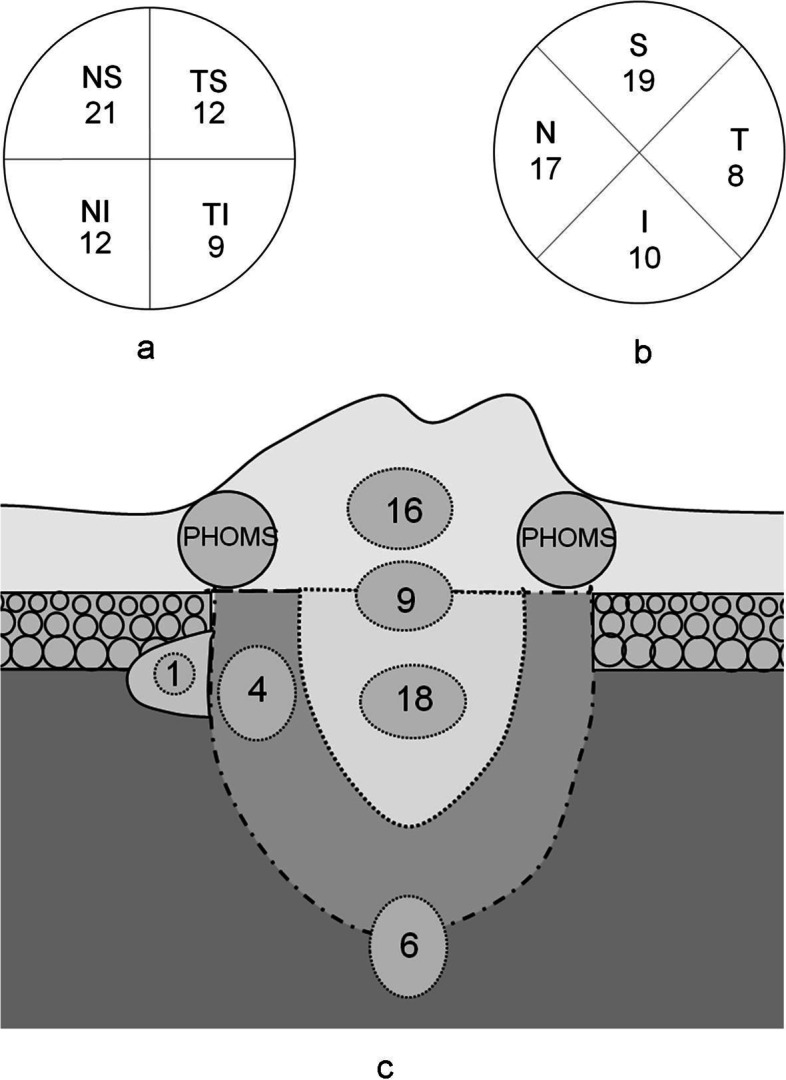
**a** and **b** The number and the orientation of ODD with regard to optic disc. **c** The schematic diagram of the location of the ODD with regard to BMO. The dotted circle showed the ODD. NS = nasal superior; NI = nasal inferior; TS = temporal superior; TI = temporal inferior; S = superior; I = inferior; N = nasal; T = temporal; PHOMS = peripapillary hyperreflective ovoid mass-like structure; BMO = Bruch’s membrane opening

**Fig. 6:**
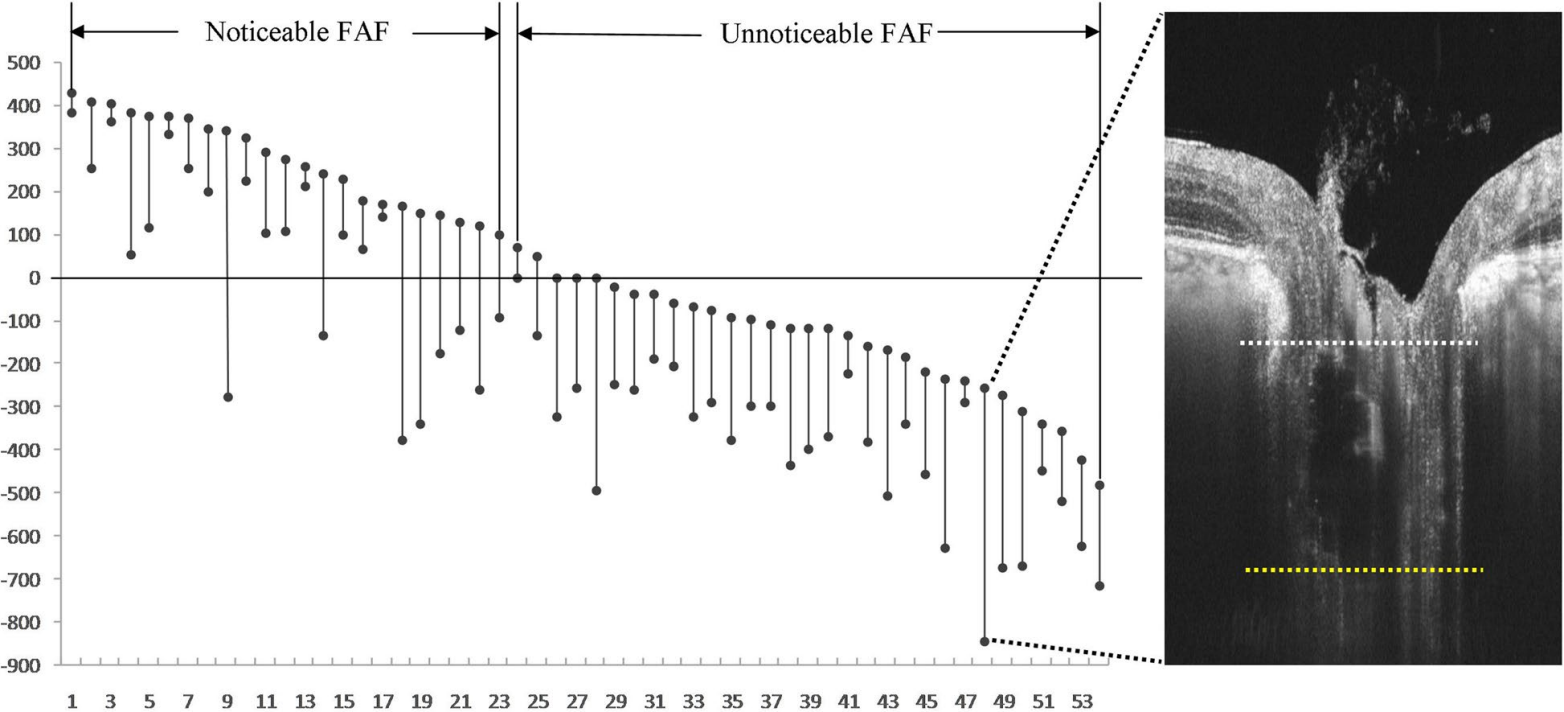
The vertical size of the ODD, and their relative location to the BMO. The length of the line represented the height of the ODD. The upper and lower point in the line represented the anterior and posterior margins of the ODD respectively. The 0 line on the y-axis represented the reference BMO. Data were arranged in a descending order according to the distance from the anterior margin of ODD to the BMO. The first 23 ODD showed autofluorescence and the distance between the anterior margin to the BMO was greater than 100 μm. While the last 31 ODD did not present noticeable autofluorescence. The distance between the posterior margin of the deepest ODD and the BMO was -844.6 μm. The anterior and posterior boundaries of the ODD were readily displayed by SS-OCT. The white dotted line was the anterior surface and the yellow dotted line was the posterior surface of the ODD

Location and size: Of the 54 ODDs in 32 eyes, SS-OCT showed 21 ODDs (38.9%) were in the nasal superior (NS), 12 (22.2%) in the nasal inferior (NI), 12 (22.2%) in the temporal superior (TS), and 9 (16.7%) in the temporal inferior (TI) of the optic disc (Fig. 5a). 19 ODDs (35.2%) were in the superior (S), 10 (18.5%) in the inferior (I), 17 (31.5%) in the nasal (N), and 8 (14.8%) in the temporal (T) of the optic disc (Fig. 5b). Seven (13.0%) were located outside the optic disc and below the BMO. One of them (1.9%) had a weak signal core in the border tissue of Elschnig (Fig. 1h, i), while other 6 (11.1%) were relatively deeper located that we suspected that they might span across the lamina cribrosa (Fig. 6). There were 47 ODDs (87.0%) located in the optic disc, of which 16 (29.6%) were above BMO, and their signal poor cores were all located in the neuroepithelial layer of the optic disc (Figs. 1e and 2d). Nine ODDs (16.7%) spanned across BMO, and their signal poor cores were located near the center of the optic disc (Fig. 2e, f). Eighteen ODDs (33.3%) were located below BMO, and their signal poor cores were also near the center of the optic disc (Fig. 1f). Finally, four ODDs (7.4%) were below BMO, and their signal poor cores were within the optic disc rim (Fig. 3e, f). The relationship between ODD and the optic disc as well as BMO was summarized in Fig. 5. The distance between the anterior margin of an ODD and BMO ranged from 431.2 to -478.0 μm (32.1 μm on average). The distance between the posterior margin of an ODD and BMO ranged from 386.7 to -844.6.0 μm (-202.9 μm on average). The maximum transverse diameter of ODD in the horizontal-scanning direction ranged from 35.3 μm to 648.1 μm (219.5 μm on average). The size of the ODD at different positions were shown in Table 1.

**Table 1:** The size of ODD and the relationship between ODD and BMO

	Above BMO	Across BMO	Below BMO
The number of ODD	16	9	29
The distance between the anterior margin of ODD and BMO (μm)	308.6 ± 98.7	162.6 ± 81.3	-161.0 ± 127.3
The distance between the posterior margin of ODD and BMO (μm)	184.5 ± 111.5	-210.5 ± 97.4	-414.5 ± 168.6
Average ODD height (μm)	124.1 ± 81.5	373.1 ± 145.2	253.4 ± 115.3
Average ODD transverse diameter (μm)	118.8 ± 63.6	275.3 ± 159.6	264.6 ± 89.4

*ODD* Optic disc drusen, *BMO* Bruch’s membrane opening

### Imaging characteristics of ODD patients on FAF

Of the 33 eyes, 9 (27.3%) superficial ODDs showed visible autofluorescence (Figs. 1b and 2b), while 24 (72.7%) buried ODDs did not (Figs. 3b and 4b). Of the 54 ODDs, when the anterior margin was ≥ 100 μm from Bruch’s membrane, clear autofluorescence could be seen. The shallower the ODD, the brighter the autofluorescence (Fig. 6).

## Discussion

Identification of ODD is of great significance in the diagnosis and differential diagnosis in eye diseases associated with optic disc. Because ODD resemble optic disc edema (ODE) in appearance, their diagnosis and assessment have been challenging. The earliest imaging modality in the diagnosis of ODD is B-scan ultrasonography, which is still the most reliable means of detecting this condition [7, 8]. In recent years, due to the advancement of technologies, much more detailed anatomical structure of ODD, as well as their association with surrounding tissues, were obtained by multimodal imaging techniques. It is therefore desirable to describe ODD based on these new findings.

It is reported that 62 ~ 76% of ODD are bilateral, emphasizing that bilaterality is a feature of ODD [9, 10]. ODD are more often located on the nasal side of the optic disc [3, 11], and our results are consistent with this feature. ODD can be superficial or buried in the optic disc. According to the relative position between ODD and BMO, we classified ODD into three categories: ODD above BMO, ODD across BMO, and ODD below BMO.

ODD above BMO usually had a pale-yellow deposit-like lesion, keeping with the traditional description of superficial ODD. Both ODD across and below BMO were not detectable by fundus photograph, which corresponded to the traditional buried ODD. By measuring the depth and transverse diameter of ODD, we found that those across BMO were the largest, followed by ODD below BMO, and those above BMO were smallest. Compared with the traditional method, classification with the relatively stable anatomic mark BMO introduced in this study had advantages of accuracy and easy for quantification, which was practical for clinical measurement and could be used to observe development and outcomes of ODD.

With the increasing popularity of OCT technology, understanding towards ODD has been improved dramatically. By increasing scanning depth with infrared laser, SS-OCT enabled us to visualize the posterior margins of ODD and observe their localization in the optic disc more accurately. In this study, the OCT features of ODD which were above or across BMO were similar to those of previous studies. Furthermore, we found that the buried ODD, which were below the BMO, could also be located within the optic disc rim or the border tissue of Elschnig. To our knowledge, such findings have not been documented before. To verify the finding, we applied 33 line scans, with the scanning lines of 6 mm in length and 2 mm in width for horizontal and vertical scan respectively. We also did cube scanning. We inspected the location of the ODD very carefully and to the best of our knowledge, excluded the possibilities of other echo, such as calcification. We suggested that the novel finding of ODD in this position was because of the SS-OCT we used. While most of the previous literatures used EDI-OCT, we used a SS-OCT which presented stronger penetrability and displayed the structure of deep tissues more clearly.

By measuring the posterior margins of ODD, we also evaluated the depth of ODD. We found that six ODDs were deeply located below the lamina cribrosa. A recent study showed that in normal subjects, the depth of central lamina cribrosa (from the anterior lamina cribrosa to the BMO) was from 209 μm to 772 μm, (402 μm on average) [12]. In current study, the distance between the posterior margin of the six deeply buried ODDs and the BMO ranged from -623.4 to -844.6 μm (-690.7 μm on average). Therefore, we speculated that the ODDs might span across the lamina cribrosa. In most cases of disc oedema and pseudooedema, the lamina cribrosa is difficult to visualize, but the ODD with high reflectivity margins could been seen at the level of the described depth of lamina cribrosa [6]. In the existing literature till date,ODD has been described above lamina cribrosa. Only one study speculated that deeply buried ODD could be located adjacent to the lamina cribrosa [13]. However, this notion has not been verified in subsequent studies. Hyperreflective horizontal lines in OCT have been reported to be related to early ODD [5, 6, 14, 15], and it has been confirmed in all cases in this study. Nevertheless, the relationship between the hyperrflective horizontal lines and the progress of the ODD deserved further studies.

It is interesting that ODD resemble ODE in appearance. In a recent consensus report from the Optic Disc Drusen Studies (ODDS) Association, PHOMS have been described[14, 16]. The appearance of PHOMS on the optic disc resemble that of pseudopapilledema, making it difficult to distinguish ODD from ODE. However, PHOMS was not a characteristics of ODD, it may exist in normal, highly myopic, AION, and eyes with ODE caused by intracranial hypertension. In this study, all 21 eyes (63.6%) with evident ODE on CFP showed PHOMS on OCT. In ODD patients without PHOMS, the morphology of optic disc was normal, and there was no edema. ODD could be diagnosed by its typical appearance on SS-OCT and quickly rule out ODE-related ocular diseases such as AION. ODD patients without PHOMS were found when they underwent examinations for other conditions or when they had screen tests. In our study, there were 12 eyes with the normal disc. ODD was suspected from OCT screening tests or OCT examinations for other conditions, including preoperative examinations of eye diseases (patient in Fig. 4) or ametropia (patient in Fig. 3). This may partially explain why the incidence of ODD is higher in autopsy than in clinical practice. However, in patients with only simple PHOMS, B-scan ultrasonography does not show characteristic hyperechoic spots. Long-term follow-up studies to reveal the relationship between PHOMS and ODD are lacking.

FAF mainly originates from the A2E fluorophore in lipofuscin particles in retinal-pigment epithelium (RPE) cells. The commonly used 488-nm laser can effectively detect lipofuscin and its precursors in the retina, as well as capture the fluorescence of porphyrin [17]. Histological examination of ODD failed to detect accumulation of lipofuscin [11], but many porphyrin substances of the respiratory chain existed in mitochondria [18]. Thus, ODD might be autofluorescent due to high level of porphyrin. Superficial ODD are more likely to be excited by autofluorescence due to their location. However, autofluorescence cannot be reliably detected in deeper ODD, perhaps because it is attenuated by the tissue overlying the drusen. Previous studies found that 93% of superficial ODD, but only 12–18% of buried ODD, could be detected by autofluorescence [19, 20]. By using SS-OCT, our results showed that when the anterior margin of ODD was > 100 μm from BMO, the autofluorescence were evident. Consequently, the autofluorescence results could be predicted with the measurement of SS-OCT. However, we do not recommend using autofluorescence for the diagnosis of buried ODD.

It should be noted that in this study one ODD case diagnosed by B-scan ultrasonography did not show abnormal optic disc findings on SS-OCT, suggesting that SS-OCT cannot detect all ODD predetermined by B-scan ultrasonography. One possible explanation was that the ODD was buried relatively deeply or some other mechanism was involved.

There are some limitations in this study. First, to ensure the patients had ODD, we only selected patients with typical B-scan ultrasonography. It may be interesting to analyze in future the SS-OCT characteristics of those patients without typical B-scan ultrasonography findings. Secondly, we did not analyze the three-dimensional data of the ODD, and it could possible to add more morphological information. Then, all the cases were Chinese. We are unaware of whether there are ethnic differences. Lastly, according to the distance between the posterior surface of ODD and the BMO, we speculated that ODD might span across the lamina cribrosa, which could be a challenge to further understanding of ODD. Clearly, the relationship between the posterior surface of ODD and the lamina cribrosa deserve further investigation.

In short, SS-OCT combined with B-scan ultrasonography and FAF provided a deeper and more comprehensive understanding of the imaging characteristics of ODD and could improve their detection rate. Compared with traditional methods, the classification using BMO proposed in this study has the advantages of accuracy measurement and quantification.

## Data Availability

All authors make sure that all data and materials support the published claims and comply with field standards. The datasets used and analysed during the current study available from the corresponding author on reasonable request.
